# Identification of black plastics with terahertz time-domain spectroscopy and machine learning

**DOI:** 10.1038/s41598-023-49765-z

**Published:** 2023-12-16

**Authors:** Paweł Piotr Cielecki, Michel Hardenberg, Georgiana Amariei, Martin Lahn Henriksen, Mogens Hinge, Pernille Klarskov

**Affiliations:** 1 Terahertz Photonics, Department of Electrical and Computer Engineering, Aarhus University, Finlandsgade 22, 8200 Aarhus N, Denmark; 2 Plastic and Polymer Engineering, Department of Biological and Chemical Engineering, Aarhus University, Aabogade 40, 8200 Aarhus N, Denmark

**Keywords:** Optics and photonics, Analytical chemistry, Sustainability

## Abstract

Several optical spectroscopy and imaging techniques have already proven their ability to identify different plastic types found in household waste. However, most common optical techniques feasible for plastic sorting, struggle to measure black plastic objects due to the high absorption at visible and near-infrared wavelengths. In this study, 12 black samples of nine different materials have been characterized with Fourier-transform infrared spectroscopy (FTIR), hyperspectral imaging, and terahertz time-domain spectroscopy (THz-TDS). While FTIR validated the plastic types of the samples, the hyperspectral camera using visible and near-infrared wavelengths was challenged to measure the samples. The THz-TDS technique was successfully able to measure the samples without direct sample contact under ambient conditions. From the recorded terahertz waveforms the refractive index and absorption coefficient are extracted for all samples in the range from 0.4 to 1.0 THz. Subsequently, the obtained values were projected onto a two-dimensional map to discriminate the materials using the classifiers k-Nearest Neighbours, Bayes, and Support Vector Machines. A classification accuracy equal to unity was obtained, which proves the ability of THz-TDS to discriminate common black plastics.

## Introduction

Plastic materials are applied in vast amounts everywhere in the modern society, where a minimum of the consumer plastics are recycled into new products^1^. It has been predicted that the amount of mismanaged plastic waste can triple and reach an amount of 200 million tons per year on a global level in 2060 if no actions are taken^2^. Poor waste management eventually leads to accumulation and leakage of plastics into our environment^3–8^. Increasing the recycling of plastic waste is challenged by consumer behaviour, undeveloped collection infrastructure, complex product designs, etc^9^. Household plastic waste collected for recycling is often a complex mixture of material types that comes in a variety of sizes and colours. This raises the challenge of obtaining sufficient (> 95%) material purity for recycling in high-value products^10^.

Optical imaging and spectroscopy systems offer fast and non-contact material identification, which have already been used for plastic sorting using sources and sensors working in different spectral regimes including X-rays, Visible (Vis), and near-infrared (NIR)^11,12^. However, plastics that are dyed black are particularly challenging due to the high absorption of light at Vis and NIR wavelengths^13–16^. X-rays have the benefit of being able to penetrate most non-metallic materials including plastics of any colour, but the high photon energies often result in a spectroscopic contrast that is too low for plastic discrimination^14,17^. Techniques based on infrared light such as Fourier-transform infrared spectroscopy (FTIR) have already been shown to be able to discriminate black plastics^16^. However, their requirement of thin samples (typically tens of micrometres) or direct contact with the sample in the attenuated total reflection (ATR) version, is unfeasible in most industrial settings where plastic waste is sorted on a conveyor belt. Mid-infrared (MIR) spectrometers based on sophisticated camera technology that upconverts light to the NIR-Vis range, have been used to characterize mixed plastic waste with a fast data collection rate of 400 Hz^18^ showing a great potential for real-time plastic sorting applications.

Another potential solution is to employ spectroscopic techniques using light at terahertz (THz) frequencies i.e. from 0.1 to 30 THz corresponding to wavelengths from 10 µm to 3 mm^19,20^. Today’s commercial THz spectroscopy systems come with coherent sources that are either in continuous wave (CW) or pulsed operation. The former is typically used for single-frequency imaging and spectroscopy since they can be relatively low-cost and compact^21^. The pulsed systems commonly employ femtosecond laser technology combined with photoconductive antennas for the generation and detection of THz pulses^22^, which advances the cost and usage of the systems. However, since the pulses in these systems are close-to single cycle with sub-picosecond duration they offer a spectral bandwidth of several THz^23^. For standard commercial THz time-domain spectroscopy (THz-TDS) systems spectral information between 100 GHz and 5 THz can be obtained^24^. In THz-TDS, pulses are recorded in the time-domain after interacting with a sample. The following Fourier transform analysis provides spectral information of the recorded pulses. Several polymers and plastics have already been characterized with THz-TDS systems that measure the material’s complex dielectric function, and hence, extract optical parameters such as the refractive index and absorption coefficient^25–31^. Moreover, inline industrial solutions using THz-TDS have been demonstrated for monitoring molten polymers^32^ and elastomer extrusion processes in rubbers^33^.

Machine Learning methods have already been widely applied to data obtained with THz technology within various applications such as agriculture, biomedicine, security inspection, and materials science^34^. For imaging of plastics with THz-TDS, neural networks have been successfully used to discriminate different plastic types^35^. However, studies dedicated to the identification of black plastics of different types in the THz range remain limited. THz imaging of black plastics has been carried out using THz camera technology sensitive in the range between 84 and 96 GHz^36^, however, the narrow spectral bandwidth limits the number of plastic types that can be discriminated with this method since non-polar polymers including PE and PS show very similar spectroscopic behaviour in this THz range^25^.

The work reported here investigates twelve commercial samples of nine types of black plastics with FTIR, hyperspectral imaging, and THz-TDS to identify their plastic type. First, the plastic types were verified with FTIR spectroscopy for plastic identification. Second, samples were examined with the inline industrial hyperspectral camera operating at wavelengths from 450 to 1740 nm in reflection geometry. However, as predicted by previous studies^13–16^, the hyperspectral camera was unable to discriminate the samples. Last, the samples were investigated with a THz-TDS system for extraction of the refractive index and absorption coefficient under ambient conditions to accommodate industrial facilities. The spectral range from 0.4 to 1.0 THz was considered since the water absorption from water vapour here was insignificant but the overall spectroscopic contrast of the measured plastic types was sufficient. Unlike most spectroscopic techniques where materials are identified from spectral features such as absorption peaks, the THz-TDS measurements showed relatively flat refractive indices and monotonically increasing absorption coefficients for all plastics. Measured values of the mean refractive indices and absorption increases were used to create a 2D map showing localized clusters for each plastic type. Common machine learning classification algorithms were applied to the 2D map, where classification accuracy equal to unity was obtained and hence, all plastic types were correctly identified.

This investigation aims to endorse the potential of THz-TDS as a future inline optical technology to identify plastics found in industrial and household waste. In contrast to other optical techniques operating at Vis and NIR wavelengths, our results prove that THz-TDS can penetrate black plastics and measure their optical constants. Despite the lack of spectral features such as absorption peaks for all the plastics investigated as well as the low refractive index contrast for the non-polar plastics such as PE and PS, the combined map of mean refractive index and absorption increase enabled the plastic identification through the machine learning classification algorithms.

## Results

**Figure 1:**
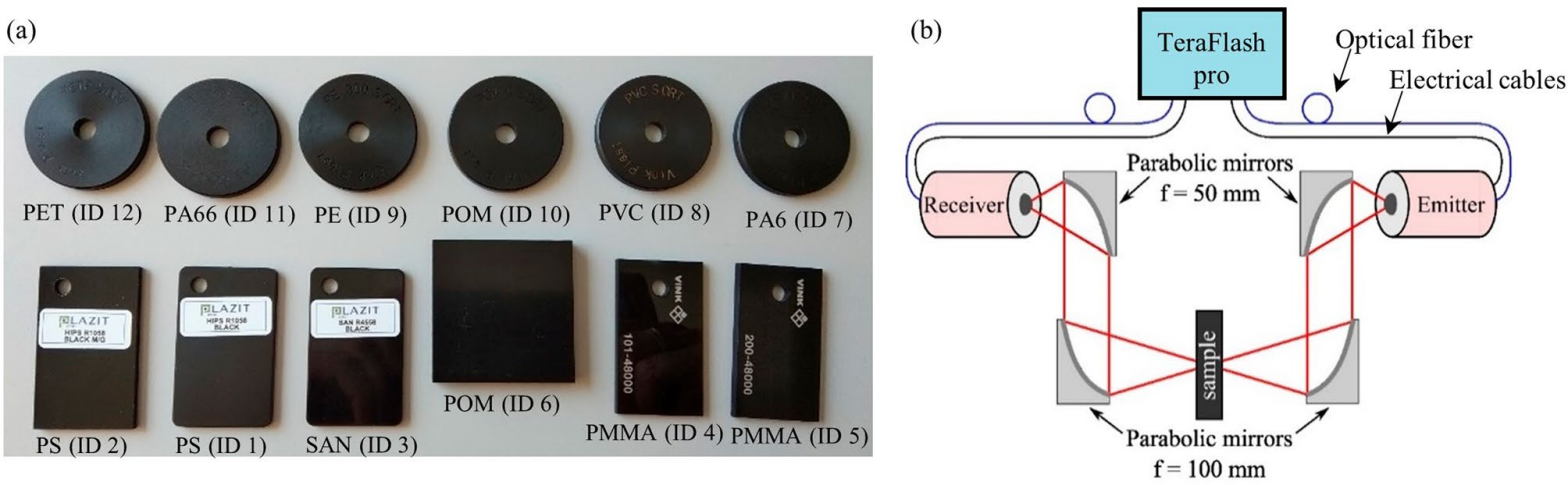
(**a**) Photo of samples and (**b**) schematic of the THz-TDS setup based on the TOPTICA TeraFlash pro system used in the study. Four off-axis parabolic mirrors are used to steer the THz beam from the fiber-coupled emitter through a sample and to the fiber-coupled receiver.

The twelve black plastics included in this study are listed in Table 1 and were used as received from the suppliers. A photo of the samples is shown in Fig. 1a.

**Table 1:** Plastic identification (ID), plastic type, trade name, and supplier for materials included in this study.

ID	Abbr.	Plastic type	Trade name	Supplier
1	PS	Polystyrene	R1058-Black	Plazit-Polygal
2	PS	Polystyrene	R1058-Black-MG	Plazit-Polygal
3	SAN	Poly(styrene-*co*-acrylonitrile)	R4558-Black	Plazit-Polygal
4	PMMA	Poly(methyl methacrylate)	101–48,000	Vink
5	PMMA	Poly(methyl methacrylate)	200–48,000	Vink
6	POM	Polyoxymethylene	SustarinC-Black	Röchling
7	PA6	Polyamide 6	PA6XTSort	Vink
8	PVC	Poly(vinyl chloride)	PVCSort	Vink
9	PE	Polyethylene	PE300Sort	Vink
10	POM	Polyoxymethylene	POMCSort	Vink
11	PA66	Polyamide 6,6	PA66-GF30^a^	Vink
12	PET	Poly(ethylene therphalate)	PETPSort	Vink

^a^Contains 30% glass fibre.

The plastic types of all samples were verified with FTIR, where individual spectra are assigned as described in Supplementary Information, section S1. Individual spectra from the hyperspectral analysis can be found in Supplementary Information, section S2. However, no spectral information could be obtained from the recorded hyperspectral spectra due to the high absorption of light at Vis and NIR wavelengths for black materials.

**Figure 2:**
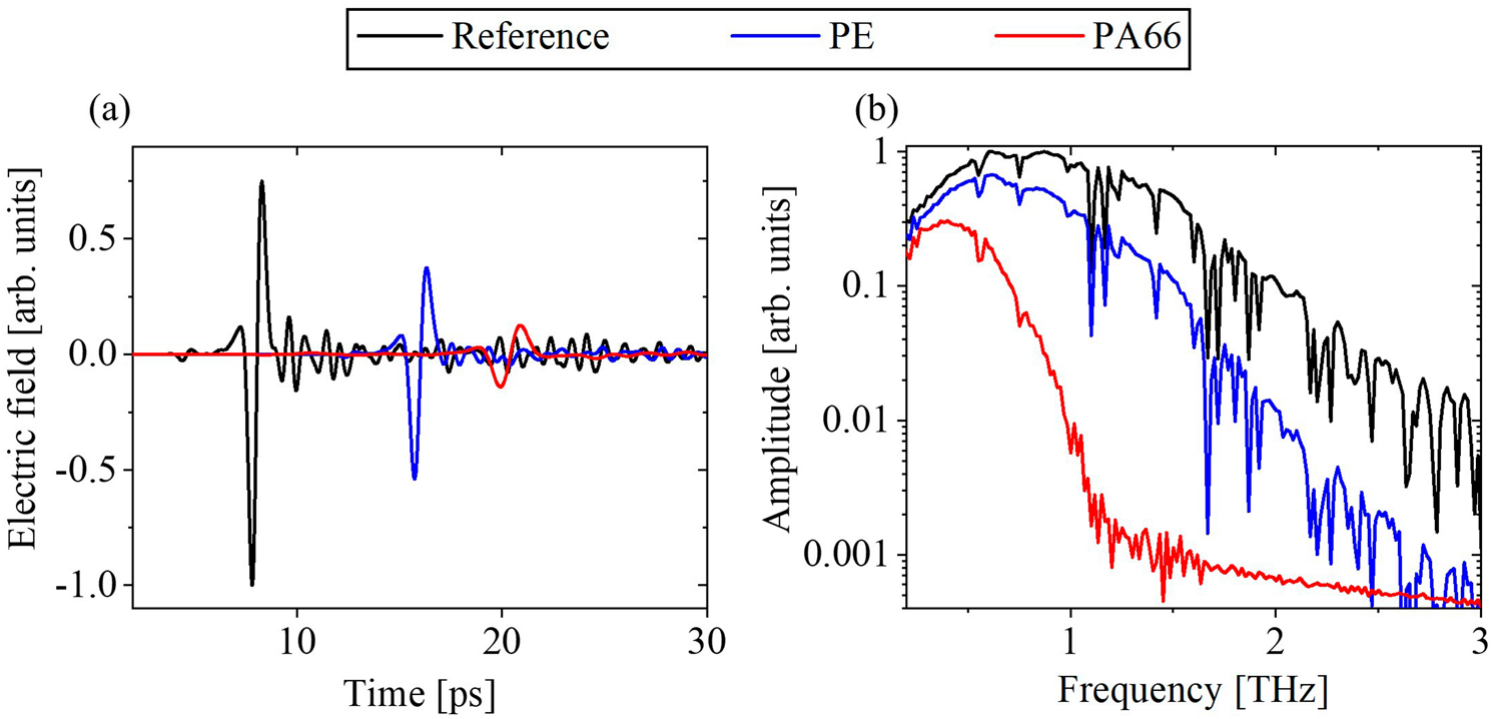
(**a**) Recorded THz-TDS waveforms for the reference measurement and samples of PE and PA66, and (**b**) corresponding spectra.

All samples were measured with a standard commercial THz-TDS setup as shown in Fig. 1b and as described under Methods. Examples of the time traces recorded through air (Reference), and the PE (ID 9) and PA66 (ID 11) samples are shown in Fig. 2a. Their corresponding amplitude spectra are shown in Fig. 2b. The reference signal (black trace) obtains the highest electric field at the earliest time position. Signals propagated through a sample are shifted in time and have a reduced amplitude due to the refractive index, absorption, and thickness of the sample. The signals recorded for PE and PA66 are shown as blue and red traces, respectively. The amplitude spectra are obtained by applying a Fourier transform to the time traces. In Fig. 2b it is seen that although our THz-TDS spectrometer covers frequencies up to at least 3 THz under ambient conditions (black trace), the higher frequencies above 1 THz are absorbed in the samples, which particularly was the case for PA66 (red trace). In the frequency range between 0.4 and 1.0 THz, the amplitude is well above the noise floor for all samples, and hence, this range is considered for the following extraction of refractive indices and absorption coefficients. This is both due to the higher transparency of the samples in this frequency range, but also because the water absorption under ambient conditions is less dominating here^37,38^. The water absorption peaks are seen as dips at 0.56 THz, 0.75 THz, 0.99 THz, 1.10 THz, 1.16 THz, etc. in Fig. 2b.

Material properties of the samples, namely refractive index (*n*) and absorption coefficient (*α*), were obtained from the measured transmission function of a sample and a reference measurement^20,39^

1 $$\begin{array}{c}\widetilde{T}\left(f\right)=\frac{{\widetilde{E}}_{S}\left(f\right)}{{\widetilde{E}}_{R}\left(f\right)}=\left|T\right|{e}^{i\Delta \phi }\end{array}$$

where ${\widetilde{E}}_{S}\left(f\right)$ is the Fourier-transformed time trace recorded through the sample, ${\widetilde{E}}_{R}\left(f\right)$ is the Fourier-transformed reference time trace recorded through air,$\left|T\right|$ is the transmission amplitude and $\Delta \phi$ is the frequency-resolved phase difference between the two signals. It was assumed that the signals propagated in the direction normal to the sample surface. The phase difference was corrected through phase unwrapping and extrapolation as described in^20^, after which the frequency-dependent refractive index was calculated as

2 $$\begin{array}{c}n\left(f\right)=1+\frac{c\Delta \phi }{2\pi fd}\end{array}$$

where $c$ is the speed of light in vacuum and $d$ is the sample thickness. For precise determination of the sample thickness, each sample was measured at 5 random places using an external digital micrometre, where the average thickness was used in the data analysis. The measured thicknesses and standard deviations are listed in Table 2.

**Table 2:** Plastic identification (ID), plastic type (abbreviation), average thickness (*d*) and standard deviation for all samples.

ID	Abbr.	Thickness, *d* [mm]	Standard deviation [mm]
1	PS	2.711	0.013
2	PS	2.989	0.001
3	SAN	2.638	0.011
4	PMMA	2.717	0.007
5	PMMA	2.934	0.001
6	POM	5.992	0.006
7	PA6	4.073	0.002
8	PVC	4.063	0.001
9	PE	4.034	0.014
10	POM	4.063	0.001
11	PA66	4.045	0.002
12	PET	4.069	0.001

**Figure 3:**
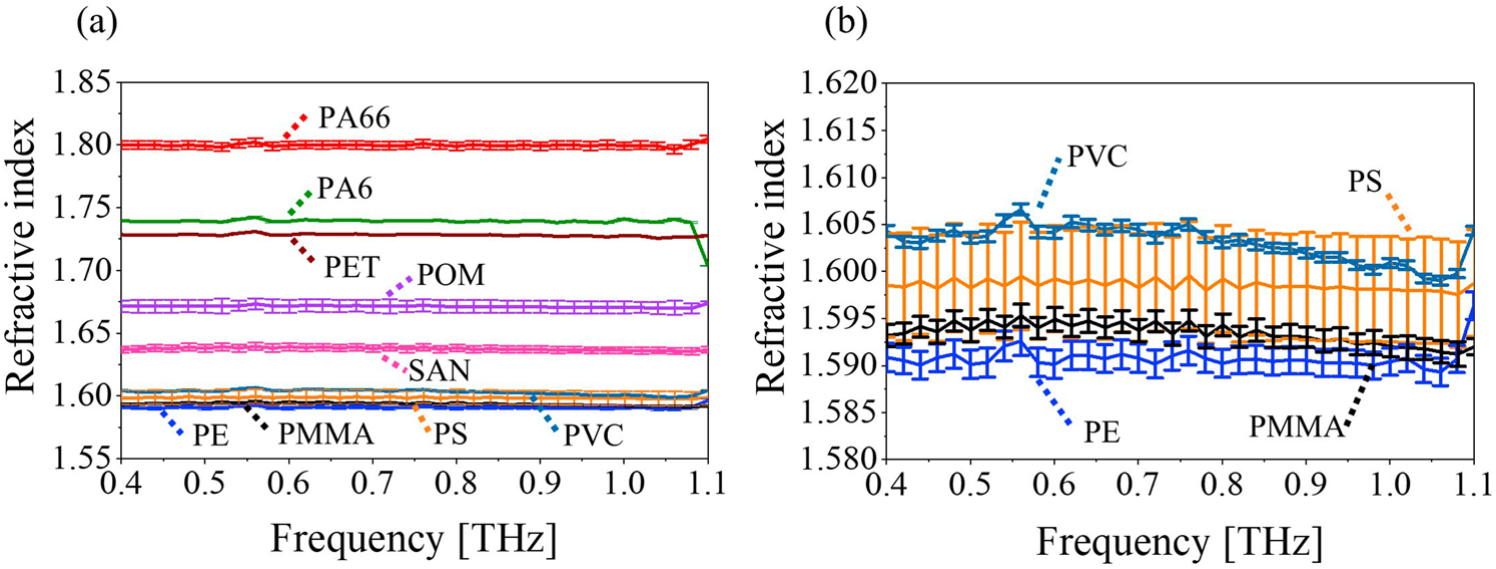
(**a**) Measured refractive indices of all materials and (**b**) zoom of the refractive indices or PS, PMMA, PVC, and PE.

Figure 3a shows the refractive index obtained from Eq. (2) for all samples. The curves are labelled with the plastic type verified by FTIR. The included errorbars are calculated from the standard deviation of ten measured time traces at different locations on the sample. For the plastic types where two different samples were measured (PS, PMMA, and POM), the curves are an average of both samples. The refractive indices of the samples PS, PMMA, PVC, and PE are slightly overlapping, so Fig. 3b shows a zoom of these. The refractive indices of the remaining materials are well separated i.e. different values are obtained for each material in the frequency range from 0.4 to 1.0 THz. Additionally, Fig. 3 shows that all materials have an almost constant value of the refractive index in this frequency range due to their low material dispersion.

With the refractive index and the transmission amplitude, the absorption coefficient is calculated by

3 $$\begin{array}{c}\alpha \left(f\right)=-\frac{2}{d}{\text{ln}}\left(\frac{{\left(n+1\right)}^{2}}{4n}\left|T\right|\right).\end{array}$$

**Figure 4:**
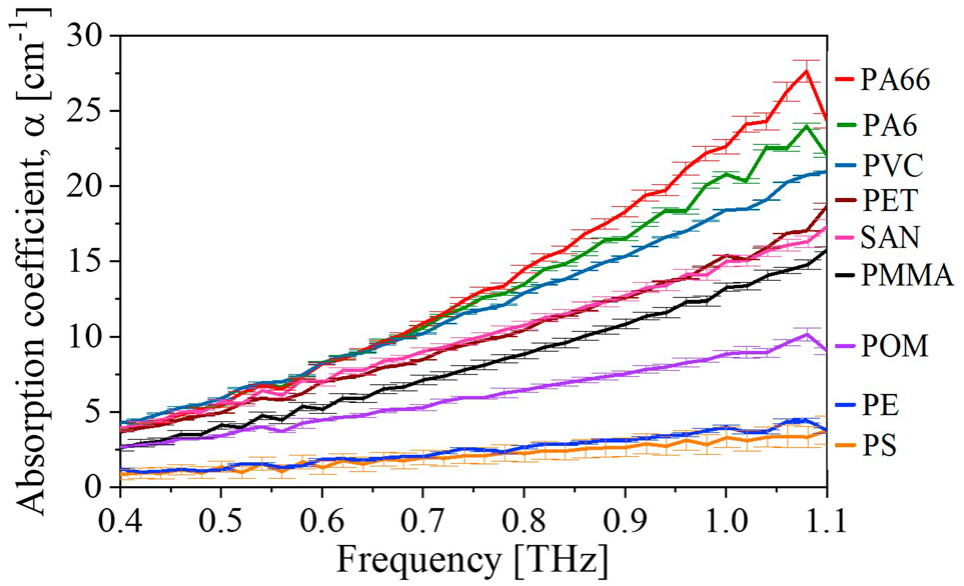
Measured absorption coefficient for all materials.

The absorption coefficients calculated from Eq. (3) are shown in Fig. 4. All materials show an overall monotonic absorption increase in the range between 0.4 and 1.0 THz. As for the refractive index, the errorbars are the standard deviation obtained from ten measurements performed on each sample.

**Figure 5:**
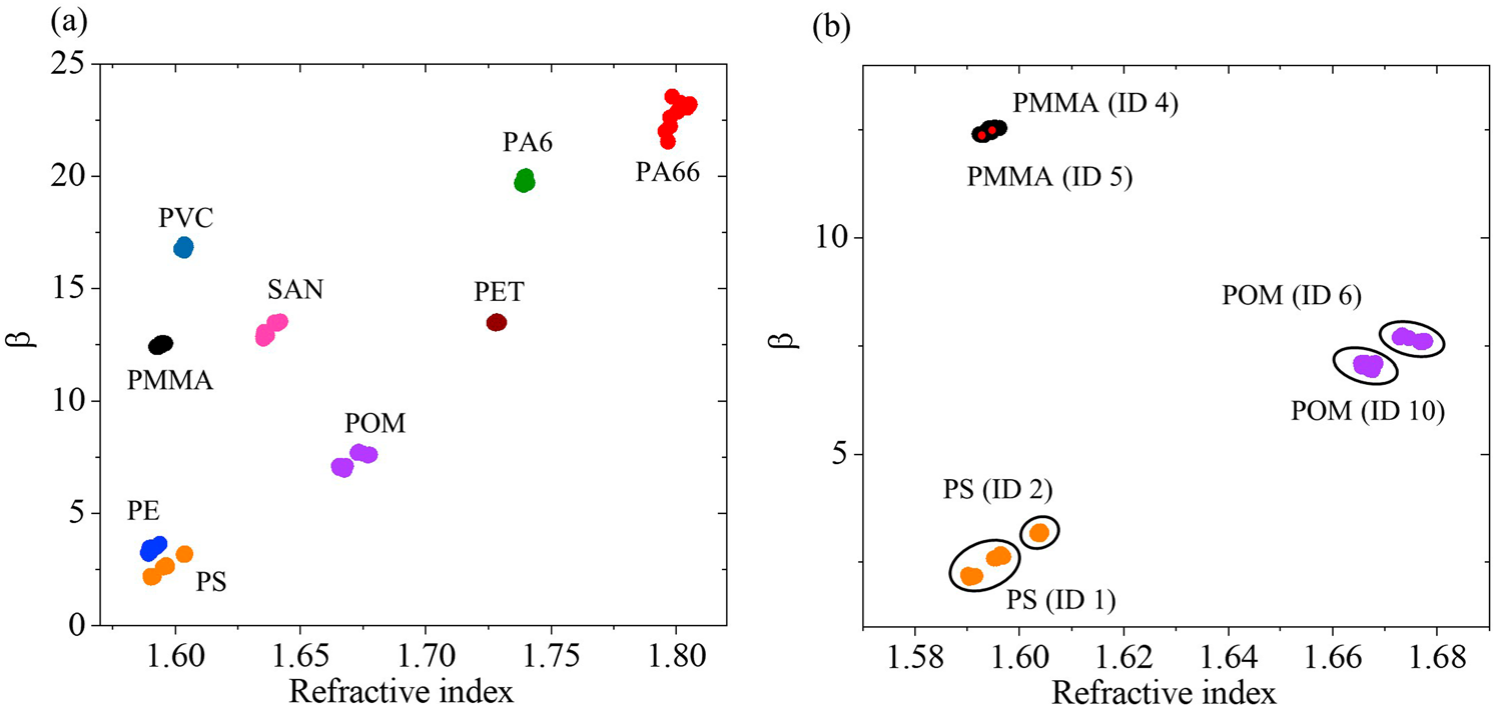
(**a**) Two-dimensional map showing the fitting parameter, $\beta$, and the refractive index of the measured materials, and (**b**) a dedicated map of PS (orange), PMMA (black) and POM (purple) that all are represented by two different samples. Black circles indicate the sample ID as indicated in Tables 1 and 2. Red dots in the PMMA cluster indicate mean values for each sample as labelled next to the cluster.

To classify the materials based on the data obtained with THz-TDS, the refractive indices and absorption coefficients were projected onto a two-dimensional space. Since the refractive index is relatively constant in the featured spectral range, its mean value is used as the first dimension. The second dimension was obtained from second-order polynomial fits to the measured absorption coefficients in the range from 0.4 to 1.0 THz. The polynomial fits are on the form

4 $$\begin{array}{c}\alpha \left(f\right)=\beta {f}^{2}+{\alpha }_{0}\end{array}$$

where the fitting parameter, $\beta$, represents the absorption increase and spans the second dimension in the two-dimensional map. ${\alpha }_{0}$ is a free fitting parameter that allows for an offset at $f=0 \; \text{Hz}$ as the trends of the absorption coefficients at frequencies below 0.4 THz are here unknown but expected to increase^28^. All fits and the obtained fitting parameters are provided in Supplementary Information section S3. The extracted values of the refractive index and the fitting parameter, $\beta ,$ are shown in Fig. 5a for all measurements. The plastic types (abbreviations) are indicated next to the clusters. Figure 5b shows a zoom of the three materials (PMMA, PS, and POM) where two different samples were measured for each material. For PS and POM, the data are split into several localized clusters, and these were identified to originate from specific samples as indicated with black circles around the clusters. For PMMA only a single cluster was observed. The small red dots on the PMMA cluster are the two slightly different mean values found for each sample ID as indicated on the map.

To classify the materials, the three classification algorithms k-Nearest Neighbours (k-NN), Bayes classifier, and support vector machines (SVM) were applied to the data shown in Fig. 5a. For all three methods, a classification accuracy of 1.0 was obtained for both the training and test sets.

## Discussion

All plastic types were successfully validated with FTIR and measured with THz-TDS, however, characterization with the hyperspectral camera at Vis and NIR wavelengths was not possible due to the high absorption in this wavelength range.

With a standard THz-TDS system, all samples were measured in the range of the system from approx. 0.1 THz to 3 THz. Since the THz range is affected by water absorption lines (see the sharp dips in the spectra in Fig. 3b), it is common practice to perform THz-TDS measurements in a dry or purged chamber to suppress water absorption affecting the measurements^23^. However, this is unfeasible in an industrial facility, so this study was carried out under ambient conditions. At 1.10 THz and beyond strong water absorption disturbed the data analysis (e.g. the decrease in refractive index for PA6 at 1.1 THz in Fig. 4a). Below 1.10 THz it was verified that the dynamic range offered by the used THz-TDS system was capable of providing reliable measurements of all samples by considering the maximum obtainable absorption (*α*_*max*_) as described by Jepsen and Fischer^40^. Even for PA66, which is the most absorbent sample, *α*_*max*_ was well above the obtained absorption (*α*) below 1.0 THz (see Supplementary Information, section S4). A larger spectral range up to beyond 2 THz could have been considered for less absorbent samples such as PE, but for the sake of consistency, the range from 0.4 to 1.0 THz was chosen for the classification of all plastic types. Lower frequencies to around 0.1 THz to 0.2 THz could have been included with the signal-to-noise ratio (SNR) of the THz-TDS spectrometer, however, the contrast in absorption coefficient for the different materials is low in this range, and hence, ignored.

To verify our results obtained with THz-TDS, Table 3 shows the values for the refractive index at 1.0 THz next to literature values for samples of the same materials measured at the same frequency. The visible appearance i.e. the colour of the samples used in the literature was not considered as it is not expected to affect the measurements in the THz range, and in most cases was not reported. Potential temperature fluctuations under ambient conditions (22 °C ± 2 °C in the used facility) are expected to deviate the measured refractive index on a level that is smaller than the included errorbars^25^.

**Table 3:** Comparison of the here measured refractive indices at 1.0 THz with values found in the literature.

ID	Abbr.	*n* (measured at 1.0 THz)	*n* (literature values at 1.0 THz)
1 & 2	PS	1.598 ± 0.0056	1.60^25^
3	SAN	1.636 ± 0.0026	1.6^41^
4 & 5	PMMA	1.592 ± 0.0011	1.584^42^, 1.61^30^
6 & 10	POM	1.671 ± 0.0048	1.68 (at 292 K)^43^
7	PA6	1.741 ± 0.0008	1.74^44^
8	PVC	1.601 ± 0.0005	1.57 to 1.62 for PVC with plasticizer concentrations between 43.14 and 9.59%, respectively^45^
9	PE	1.590 ± 0.0015	1.59^26^
11	PA66	1.799 ± 0.0031	1.90 and 1.87 for 30% glass fibre aligned parallel and perpendicular to the THz polarization, respectively^46^, 1.74 (unknown glass fibre content)^44^
12	PET	1.728 ± 0.0006	1.712^47^, 1.69 and 1.73 for amorphous and semi-crystalline PET, respectively^48^

Table 3 shows that the obtained refractive indices for PS, SAN, POM, PA6, PE, and PET agree with the literature values. For PVC, a good agreement is found when considering the refractive indices of samples measured with concentrations of plasticizers in the range from 10 to 43%^45^. Although the plasticizer content of our sample is unknown, it is expected to be in the lower part of this range as this is most commonly used in PVC^49^. The measured refractive index of 1.592 ± 0.0011 for PMMA falls between the two reported values of 1.584 and 1.61 facilitating the tendency of variation in the literature values for the specific plastic types.

The measured refractive index for PA66 with 30% glass fibre of 1.799 ± 0.0031 at 1.0 THz is somewhat lower than the literature values of 1.90 and 1.87 found for the same material using THz pulses polarized parallel and perpendicular to the direction of the fibres, respectively^46^. As no polarization difference was observed for our sample, the fibres are expected to be randomly oriented. The deviation is expected to be due to the inhomogeneously distributed fibres, which is implied in the relatively large standard deviation of 0.0031 for this sample. A much lower value of 1.74 for PA66 was reported by Piesiewicz et al*.*^44^, and although no glass fibre content was stated here, the varying refractive indices may be expected for such samples where the homogeneity and purity are unknown.

Deviations of the refractive indices are mainly expected to be due to sample variations originating from contaminants or uneven thicknesses. This is primarily seen for POM and PS where two samples for each plastic type were investigated, which corresponded to distinct sub-clusters in Fig. 5b. For PMMA the measurements of the two different samples were confined to a single cluster. For all materials, including POM and PS, the clusters for each plastic type were well separated from each other meaning that material separation easily could be obtained by considering Fig. 5a. Material identification was further manifested by the machine learning algorithms k-NN, Bayes classifier, and SVM, where a classification accuracy equal to unity for both training and test sets was obtained. It is noted that in the context of machine learning, the amount of data used here i.e. 120 data points obtained for nine different materials as shown in Fig. 5a is relatively small, and future studies may consider more samples of the same materials yielding a larger number of total measurements to challenge the classification algorithms. However, the distinct clusters and perfect classification are considered as proofs of the ability to discriminate the studied types of black plastics using THz-TDS for automated identification of plastics.

The THz-TDS technique is here used in a transmission geometry, where the obtained refractive index and absorption coefficient represent the material of the entire thickness of the sample where it is measured. This is in contrast to many other optical techniques such as NIR and Vis hyperspectral imaging that are carried out in reflection geometry and only measure the material at the surface of the sample. The measured values of refractive index and absorption represent an average value of the investigated material and rely on the sample thickness, which was separately measured with a micrometre. This is unfeasible in an industrial context where random pieces of plastic waste may need to be sorted on a conveyor belt. However, THz-TDS can be carried out in a reflection geometry, and it has been proven possible to simultaneously measure the refractive index and thickness of a sample of silicon by including both the THz pulses reflected from the frontside and backside of the sample^50^. Recently, a new high-speed THz-TDS system providing 150 ps time traces at the rate of 1600 traces/s has been used to image a metallic structure reflection geometry^51^. Each linescan of the 500 mm long moving metallic structure was measured in 1.4 s corresponding to a measurement speed of > 350 mm/s with a resolution of 0.44 mm. This speed is superior to the conveyor belt speed of 62.5 mm/s under the commercial hyperspectral camera setup, which is intended for industrial use^12^. Further developments in fast reflection geometry measurements together with the results presented in this report emphasize that THz-TDS has the potential to be implemented as a plastic type identification tool for black plastics found in industrial and household waste in the future.

## Conclusion

In this study, 12 black samples of nine different types of materials have been studied with the three optical techniques: FTIR, hyperspectral imaging, and THz-TDS. FTIR was able to validate the material types of the samples, while the hyperspectral camera was unable to measure the samples due to the high material absorption in the spectral range of Vis and NIR wavelengths. The THz-TDS technique was successfully able to measure and discriminate the samples under ambient conditions through the extraction of the refractive indices and absorption coefficients. Machine learning algorithms based on k-NN, Bayes, and SVM were used to classify the materials through the measured refractive indices and absorption coefficients in the spectral range from 0.4 to 1.0 THz. A classification accuracy equal to unity was obtained for both test and training sets of the data with a fivefold cross-validation. This proves that THz-TDS can discriminate the most common household plastic types, even for black materials that most other optical techniques struggle to measure.

## Methods

### Fourier transform infrared spectroscopy

ATR FTIR spectra of the samples were collected with an infrared spectrophotometer using a ZnSe crystal. Background and sample spectra were measured with a resolution of 2 cm^−1^, both recorded with 16 scans per measurement. Wavelength-dependent penetration depth and baseline were corrected with built-in functions of OMNIC (v. 9.2.98., Thermo Scientific, USA) prior to the analysis.

### Hyperspectral camera analysis

The hyperspectral camera setup analysis was performed using a commercial setup from Newtec A/S. It has a 29 cm wide conveyor belt for transportation of samples with a speed of 62.5 mm/s under two line-scan hyperspectral cameras (Oculus QT5022 detectors, Buteo Vis and Buteo SWIR, Qtechnology, DK). Samples were illuminated at 45° by two rows of four halogen spots (12 V, 20 W). Prior to measurement, a full calibration was performed^52^. Intensity calibration was referenced to TiO_2_. The spatial resolution was 0.22 mm by 0.5 mm and 1.1 mm by 0.5 mm across and along the conveyor belt for Vis and SWIR, respectively. The spectral resolution was 1.8 nm from 450 to 1050 nm and 9.2 nm from 955 to 1740 nm. The samples were loaded on the conveyor belt in two rows, passed the cameras, and the raw data cube was obtained. The reported spectra are a summation of 2000 spectra (100 by 20 pixels) for Vis and 400 spectra (20 by 20 pixels) for SWIR. All spectra were transformed to and reported as absorbance.

### Terahertz time-domain spectroscopy

The samples were measured with a fiber-coupled, commercial THz-TDS spectrometer manufactured by TOPTICA Photonics (TeraFlash pro). The setup was arranged in a transmission configuration using four off-axis parabolic mirrors between the fiber-coupled THz emitter and receiver (see Fig. 1b). A 50 mm focal length parabolic mirror collimates the THz radiation from the emitter, while a parabolic mirror with a focal length of 100 mm focuses it onto the sample. Likewise, a 100 mm focal length parabolic mirror collimates the THz radiation transmitted through the sample, and a 50 mm focal length parabolic mirror focuses it into the receiver. For each sample, ten measurements at random positions on the sample were recorded followed by a single reference measurement where the sample was absent. All the measurements were performed under the same ambient experimental conditions recording time traces with a length of 50 ps and 1000 acquisitions (scan speed: 60 traces/s). Before calculating the transmission function in frequency domain as described in Eq. (1), the obtained time-domain signals were artificially extended to 60 ps by zero-padding to ensure that the measured pulse is positioned before the midpoint of the time window. Failure to do so may lead to overcorrection of the phase^53^. This was the case for the sample with ID 6 (POM), which was the sample with the largest thickness of ~ 6 mm.

### Machine learning algorithms

The three common algorithms k-Nearest Neighbours (k-NN), Bayes classifier, and support vector machines (SVM) were used to classify the results in Fig. 5a. Prior to this study, these classifiers have successfully been applied to THz spectroscopy data obtained for a similar spectral range to identify explosives^54,55^.

*k-Nearest Neighbours* is a simple algorithm representative of the so-called lazy learning algorithms, where the training phase is not performed^56^. It classifies a new observation based on the majority vote of the *k* most similar training instances (nearest neighbours). The Euclidean distance was used as a measure of similarity, and a feedback of the five nearest neighbours was considered.

*Bayes Classifier* is a probabilistic model based on Bayes’ theorem^57^. The probability that the observation belongs to the specific class (posterior probability) is calculated using the prior and the likelihood, which is estimated from the training data. The prior is the probability that the observation belongs to the specific class, while the likelihood is the probability that the observation with the given values belongs to that specific class. The class likelihood function was assumed to be a multivariate Gaussian distribution, and hence, the parameters required for the estimation were limited to the mean vector and the covariance matrix. Quadratic discriminant analysis (QDA) was performed to allow the covariance matrix to vary between the classes.

*Support Vector Machines* developed in 1995 by Vapnik is one of the most robust and most commonly used classification algorithms^58^. It aims to find a hyperplane that separates two classes with the largest margin, which is the minimum geometrical distance to class representatives. In this study, the slack variable, which is used in a soft margin approach (where some observations are allowed to violate the margin), was determined via a fivefold cross-validation of the training data. The multiclass classification problem was solved by dividing it into multiple binary problems using a one-versus-one approach. Here, a linear kernel was applied in the SVM model.

The classification accuracy of the relatively small dataset obtained here was enhanced by using fivefold cross-validation^59,60^. In this method, the data is partitioned into five equally sized groups using stratified random sampling. Stratified implies that each partition is a good representation of the entire dataset. A partition is first selected as a test set, while the four remaining partitions are used for training. This process is iteratively repeated for all partitions to constitute a test set i.e. five times, and the classification accuracy is an average over all folds.

### Supplementary Information

Supplementary Information.

## Data Availability

The datasets used are available from the corresponding author P. K. on request.

## References

[1] Industry Agenda: The new plastics economy rethinking the future of plastics. Paper presented at *World Economic Forum*, Geneva, Switzerland (2016).

[2] Lebreton L, Andrady A (2019). Future scenarios of global plastic waste generation and disposal. Palgrave Commun..

[3] Barnes DK, Walters A, Goncalves L (2010). Macroplastics at sea around Antarctica. Mar. Environ. Res..

[4] Colferai AS, Silva-Filho RP, Martins AM, Bugoni L (2017). Distribution pattern of anthropogenic marine debris along the gastrointestinal tract of green turtles (*Chelonia mydas*) as implications for rehabilitation. Mar. Pollut. Bull..

[5] Corcoran PL, Biesinger MC, Grifi M (2009). Plastics and beaches: A degrading relationship. Mar. Pollut. Bull..

[6] Martinez E, Maamaatuaiahutapu K, Taillandier V (2009). Floating marine debris surface drift: Convergence and accumulation toward the South Pacific subtropical gyre. Mar. Pollut. Bull..

[7] Ryan PG, Moore CJ, van Franeker JA, Moloney CL (2009). Monitoring the abundance of plastic debris in the marine environment. Philos. Trans. R. Soc. Lond. B Biol. Sci..

[8] Thompson RC (2004). Lost at sea: Where is all the plastic?. Science.

[9] Matthews C, Moran F, Jaiswal AK (2021). A review on European Union’s strategy for plastics in a circular economy and its impact on food safety. J. Clean. Prod..

[10] Alassali A (2021). Towards higher quality of recycled plastics: Limitations from the material’s perspective. Sustainability.

[11] Lubongo C, Alexandridis P (2022). Assessment of performance and challenges in use of commercial automated sorting technology for plastic waste. Recycling.

[12] Henriksen ML, Karlsen CB, Klarskov P, Hinge M (2022). Plastic classification via in-line hyperspectral camera analysis and unsupervised machine learning. Vib. Spectrosc..

[13] Rozenstein O, Puckrin E, Adamowski J (2017). Development of a new approach based on midwave infrared spectroscopy for post-consumer black plastic waste sorting in the recycling industry. Waste Manag..

[14] Serranti, S. & Bonifazi, G. In *Use of Recycled Plastics in Eco-efficient Concrete* (eds Pacheco-Torgal, F. *et al.*) 9–37 (Woodhead Publishing, 2019).

[15] Bonifazi G, Capobianco G, Serranti S (2018). A hierarchical classification approach for recognition of low-density (LDPE) and high-density polyethylene (HDPE) in mixed plastic waste based on short-wave infrared (SWIR) hyperspectral imaging. Spectrochim. Acta A Mol. Biomol. Spectrosc..

[16] Signoret C, Caro-Bretelle AS, Lopez-Cuesta JM, Ienny P, Perrin D (2019). MIR spectral characterization of plastic to enable discrimination in an industrial recycling context: I. Specific case of styrenic polymers. Waste Manag..

[17] Vrancken C, Longhurst PJ, Wagland ST (2017). Critical review of real-time methods for solid waste characterisation: Informing material recovery and fuel production. Waste Manag..

[18] Zinchik S (2021). Accurate characterization of mixed plastic waste using machine learning and fast infrared spectroscopy. ACS Sustain. Chem. Eng..

[19] Tonouchi M (2007). Cutting-edge terahertz technology. Nat. Photon..

[20] Jepsen PU, Cooke DG, Koch M (2011). Terahertz spectroscopy and imaging—Modern techniques and applications. Laser Photonics Rev..

[21] Karpowicz N (2005). Comparison between pulsed terahertz time-domain imaging and continuous wave terahertz imaging. Semicond. Sci. Technol..

[22] Fattinger C, Grischkowsky D (1989). Terahertz beams. Appl. Phys. Lett..

[23] Neu J, Schmuttenmaer CA (2018). Tutorial: An introduction to terahertz time domain spectroscopy (THz-TDS). J. Appl. Phys..

[24] Naftaly M, Vieweg N, Deninger A (2019). Industrial applications of terahertz sensing: State of play. Sensors.

[25] Wietzke S (2011). Terahertz spectroscopy on polymers: A review of morphological studies. J. Mol. Struct..

[26] Cunningham PD (2011). Broadband terahertz characterization of the refractive index and absorption of some important polymeric and organic electro-optic materials. J. Appl. Phys..

[27] Busch SF (2014). Optical properties of 3D printable plastics in the THz regime and their application for 3D printed THz optics. J. Infrared Millim. Terahertz Waves.

[28] Squires AD, Lewis RA (2018). Feasibility and characterization of common and exotic filaments for use in 3D printed terahertz devices. J. Infrared Millim. Terahertz Waves.

[29] Podzorov A, Gallot G (2008). Low-loss polymers for terahertz applications. Appl. Opt..

[30] Naftaly M, Miles RE (2007). Terahertz time-domain spectroscopy for material characterization. Proc. IEEE.

[31] Nagai N, Fukasawa R (2004). Abnormal dispersion of polymer films in the THz frequency region. Chem. Phys. Lett..

[32] Krumbholz N (2009). Monitoring polymeric compounding processes inline with THz time-domain spectroscopy. Polym. Test..

[33] Peters O (2013). Terahertz spectroscopy for rubber production testing. Polym. Test..

[34] Jiang Y (2022). Machine learning and application in terahertz technology: A review on achievements and future challenges. IEEE Access.

[35] Kubiczek T, Balzer JC (2022). Material classification for terahertz images based on neural networks. IEEE Access.

[36] Küter A, Reible S, Geibig T, Nüßler D, Pohl N (2018). THz imaging for recycling of black plastics. tm Technisches Messen.

[37] Xin X, Altan H, Saint A, Matten D, Alfano RR (2006). Terahertz absorption spectrum of para and ortho water vapors at different humidities at room temperature. J. Appl. Phys..

[38] Exter MV, Fattinger C, Grischkowsky D (1989). Terahertz time-domain spectroscopy of water vapor. Opt. Lett..

[39] Withayachumnankul W, Naftaly M (2013). Fundamentals of measurement in terahertz time-domain spectroscopy. J. Infrared Millim. Terahertz Waves.

[40] Jepsen PU, Fischer BM (2005). Dynamic range in terahertz time-domain transmission and reflection spectroscopy. Opt. Lett..

[41] Mumtaz M (2017). Investigation of dielectric properties of polymers and their discrimination using terahertz time-domain spectroscopy with principal component analysis. Appl. Spectrosc..

[42] Islam MS (2020). Experimental study on glass and polymers: Determining the optimal material for potential use in terahertz technology. IEEE Access.

[43] Wietzke S (2009). Terahertz time-domain spectroscopy as a tool to monitor the glass transition in polymers. Opt. Express.

[44] Piesiewicz R (2007). Properties of building and plastic materials in the THz range. Int. J. Infrared Millim. Waves.

[45] Sommer S, Koch M, Adams A (2018). Terahertz time-domain spectroscopy of plasticized poly(vinyl chloride). Anal. Chem..

[46] Rutz, F. *et al.* Non-destructive testing of glass-fibre reinforced polymers using terahertz spectroscopy. In *9th European Conference on Non-Destructive Testing 2006,* https://www.ndt.net/?id=4050 (2006).

[47] Jin Y-S, Kim G-J, Jeon S-G (2006). Terahertz dielectric properties of polymers. J. Korean Phys. Soc..

[48] Engelbrecht S (2019). Monitoring the isothermal crystallization kinetics of PET-A using THz-TDS. J. Infrared Millim. Terahertz Waves.

[49] Chaudhary BI, Liotta CL, Cogen JM, Gilbert M (2016). Plasticized PVC. Ref. Modul. Mater. Sci. Mater. Eng..

[50] Vandrevala F, Einarsson E (2018). Decoupling substrate thickness and refractive index measurement in THz time-domain spectroscopy. Opt. Express.

[51] Palka N (2022). Fast THz-TDS reflection imaging with ECOPS-point-by-point versus line-by-line scanning. Sensors.

[52] Henriksen ML, Pedersen WN, Klarskov P, Hinge M (2022). One step calibration of industrial hyperspectral cameras. Chemom. Intell. Lab. Syst..

[53] Jepsen PU (2019). Phase retrieval in terahertz time-domain measurements: A “how to” tutorial. J. Infrared Millim. Terahertz Waves.

[54] Cielecki PP, Kristensen MH, Skovsen E (2021). Analysis and classification of frequency-domain terahertz reflection spectra using supervised and unsupervised dimensionality reduction methods. J. Infrared Millim. Terahertz Waves.

[55] Kristensen, M. H., Cielecki, P. P. & Skovsen, E. Classification of terahertz reflection spectra using machine learning algorithms. In *2022 47th International Conference on Infrared, Millimeter and Terahertz Waves (IRMMW-THz),*10.1109/IRMMW-THz50927.2022.9895909 (2022).

[56] Boiman, O., Shechtman, E. & Irani, M. In defense of nearest-neighbor based image classification. In *2008 IEEE Conference on Computer Vision and Pattern Recognition,*10.1109/CVPR.2008.4587598 (2008).

[57] Alpaydin E (2020). Introduction to Machine Learning.

[58] Cortes C, Vapnik V (1995). Support-vector networks. Mach. Learn..

[59] Shalev-Shwartz S, Ben-David S (2014). Understanding Machine Learning: From Theory to Algorithms.

[60] Yadav, S. & Shukla, S. Analysis of k-fold cross-validation over hold-out validation on colossal datasets for quality classification. In *2016 IEEE 6th International Conference on Advanced Computing (IACC),* 78–83 10.1109/IACC.2016.25 (2016).

