# Supplementary material for: Identification of black plastics with terahertz time-domain spectroscopy and machine learning
Source: Sci Rep. 2023 Dec 16;13:22399. doi: 10.1038/s41598-023-49765-z (PMC10725460; doi:10.1038/s41598-023-49765-z)
Supplement: Supplementary file 1 — Supplementary Information. [file 41598_2023_49765_MOESM1_ESM.pdf]

Supplementary Information

Identification of black plastics with terahertz time-domain spectroscopy and machine learning

Paweł Piotr Cielecki[†], Michel Hardenberg[†], Georgiana Amariei[‡], Martin Lahn Henriksen[‡], Mogens Hinge[‡], Pernille Klarskov[†]*

[†] Terahertz Photonics, Department of Electrical and Computer Engineering, Aarhus University, Finlandsgade 22, DK-8200, Aarhus N, Denmark.

[‡] Plastic and Polymer Engineering, Department of Biological and Chemical Engineering, Aarhus University, Aabogade 40, DK-8200 Aarhus N., Denmark.

* Corresponding author: Pernille Klarskov, klarskov@ece.au.dk

Content

S1. ATR FTIR spectra of all components 2

S2. Hyperspectral camera spectra of all components 5

Images of channel 90 in the hypercubes. 5

Combined spectra 6

S3. Absorption fits 7

S4. Maximum absorption 9

S1. ATR FTIR spectra of all components

Individual and detailed ATR FTIR spectra measured with FTIR for all samples spectra of all the samples are shown below.

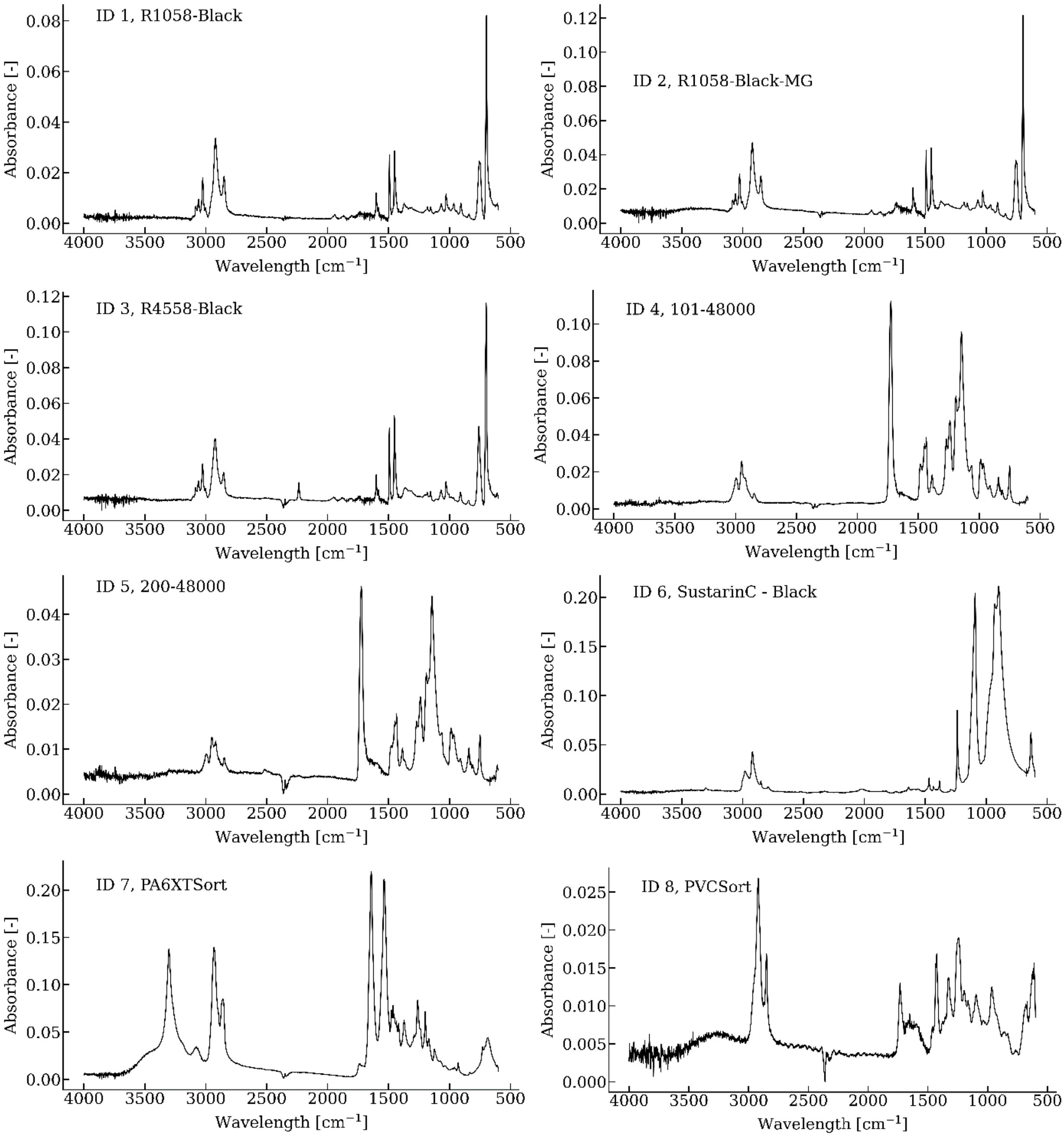

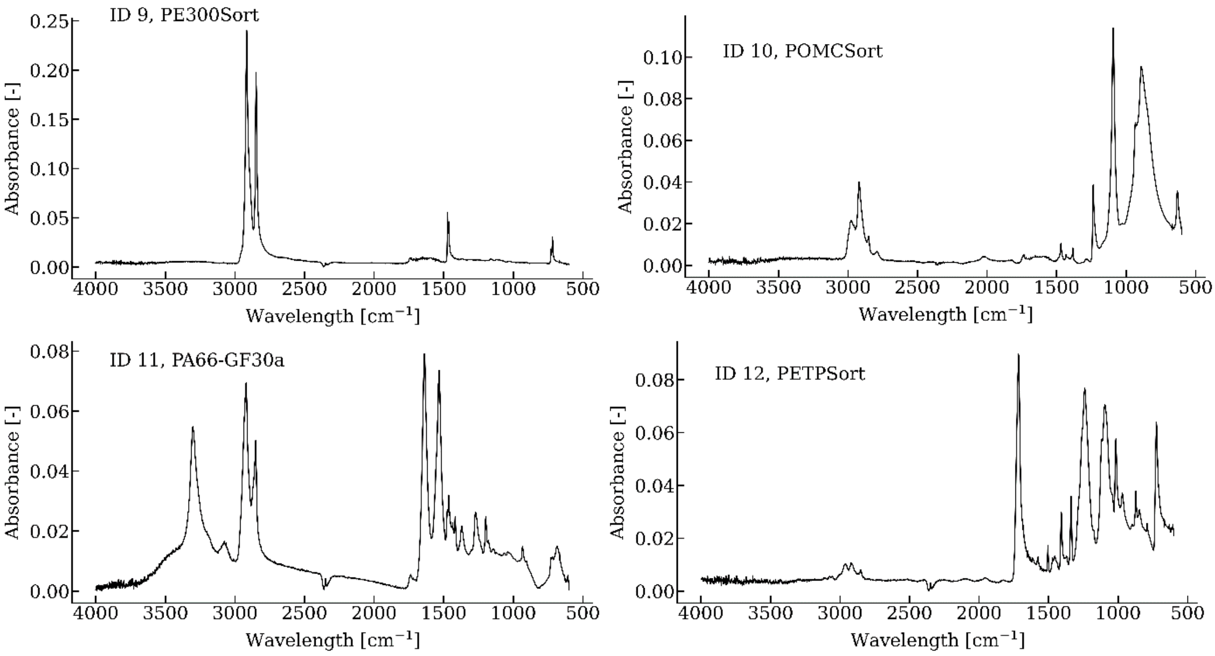

The spectra are assigned as; **PE** (ID 9): The bands at 2915 and 2850 cm^{-1} are assigned to sp^3 hybrididized CH together with stretch (^{v}) and bending ($^{\delta}$) of CH found at 1465, and 720 cm^{-1} [38]. **POM** (ID 6 and 10): Similar bands to PE and additional bands at 1235, 1090, 925, 895, and 630 cm^{-1} assigned to the ether [38, 39]. **PVC** (ID 8): Similar bands to PE (shifted down by ≈30 cm^{-1}) and additional bands at 1330, 1100, 965, and 610 cm^{-1} for CH-Cl, C-C, $CH_{2(rock)}$ and C-Cl, respectively, and the thermostabilizing additive is found at 1730, 1260 and 1185 cm^{-1} and with some overlap of PVC (bands for $^{v,\delta}CH_2$, and $^{v,\delta}$CH) [38, 40, 41]. **PA6** (ID 7): Similar backbone bands to PE and additional bands at 3300 cm^{-1} ^{v}NH and $^{\delta}$NH at 1535 cm^{-1} (with overtone at 3080 cm^{-1}) and a band at 1640 cm^{-1} ascribed to ^{v}C=O [38]. **PA66** (ID 11): Same as PA6, but with a large signal for 2920 and 2850 cm^{-1} ($^{v,\delta}CH_2$) relative to the amide band at 3300 cm^{-1}. The glass fibres are found at 800 to 1200 cm^{-1} for the Si-O-Si (peak at 1020 cm^{-1}). **PMMA** (ID 4 and 5): CH_2 and CH_3 sp^3 hybrididized around 2950 cm^{-1} and bands for CH deformation at 1435, 1144 and 749 cm^{-1}. Additional bands at 1723 cm^{-1} from ^{v}C=O, 1269, 1190, and 840 cm^{-1} from the ^{v}C-O and C-O-C assymetric stretch at 1239 cm^{-1} are evident [42]. **PS** (ID 1 and 2): Similar backbone bands to PE and additional bands for the aromatic

$^{\nu,\delta}CH_{ar}$ (and ring bend) is evident at 3080, 3060, 3025, 1600, and 1490 cm^{-1}. A monosubstituted benzene is evident from the pattern of the overtones (1650 to 1950 cm^{-1}) and bands at 755, and 695 cm^{-1}. **SAN** (ID 3): Similar bands to PS and additional at 2250 cm^{-1} from the acrylonitrile [38, 43]. **PET** (ID 12): The band at 3037 cm^{-1} is ascribed to sp^2 hybrididized CH_{ar} and sp^3 hybrididized CH is found at 2960, 2920 and 2855 cm^{-1} for the glycol part with additional bands at 1714 cm^{-1} from $^{\nu}C{=}O$ and 1238 and 1091 cm^{-1} for $^{\nu}C\text{-}O$. Finally, the band at 723 cm^{-1} for para-disubstituted benzene is found. The above assignment show that the materials are successfully verified.

S2. Hyperspectral camera spectra of all components

Combined, individual and detailed spectra, extracted from the data cube obtained from the hyperspectral camera, of all the samples in the study.

Images of channel 90 in the hypercubes.

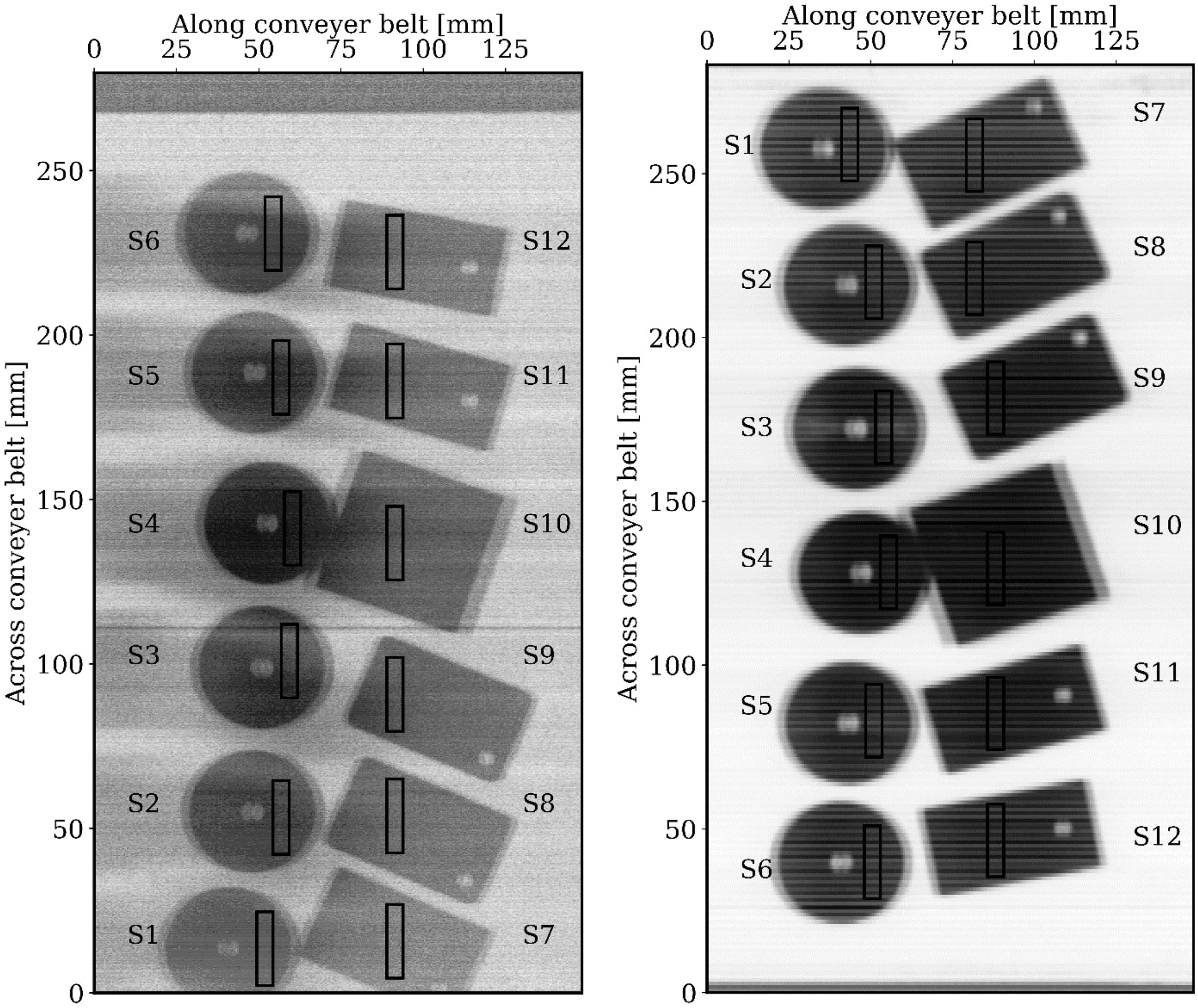

Left Vis and Right SWIR.

Sample numbering are: ID 1: S8, ID 2: S7, ID 3: S9, ID 4: S11, ID 5: S12, ID 6: S10, ID 7: S6, ID 8: S5, ID 9: S3, ID 10: S4, ID 11: S2, and ID 12: S1.

Combined spectra

Absorbance spectra for the Vis (Right) and SWIR (Left) spectra ranges are shown below

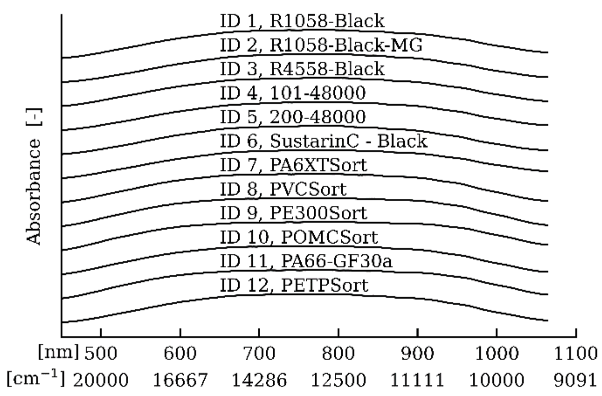

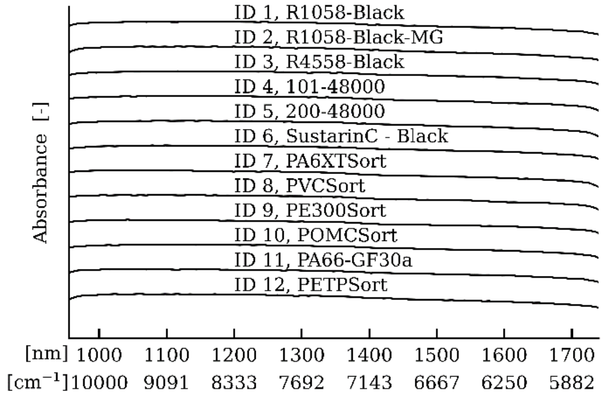

S3. Absorption fits

Below are the obtained absorption data measured with THz-TDS together with polynomial fits as described in Eq. (4). The mean R^2-values for each material are indicated on the figures (here representing both sample for PS, PMMA and POM).

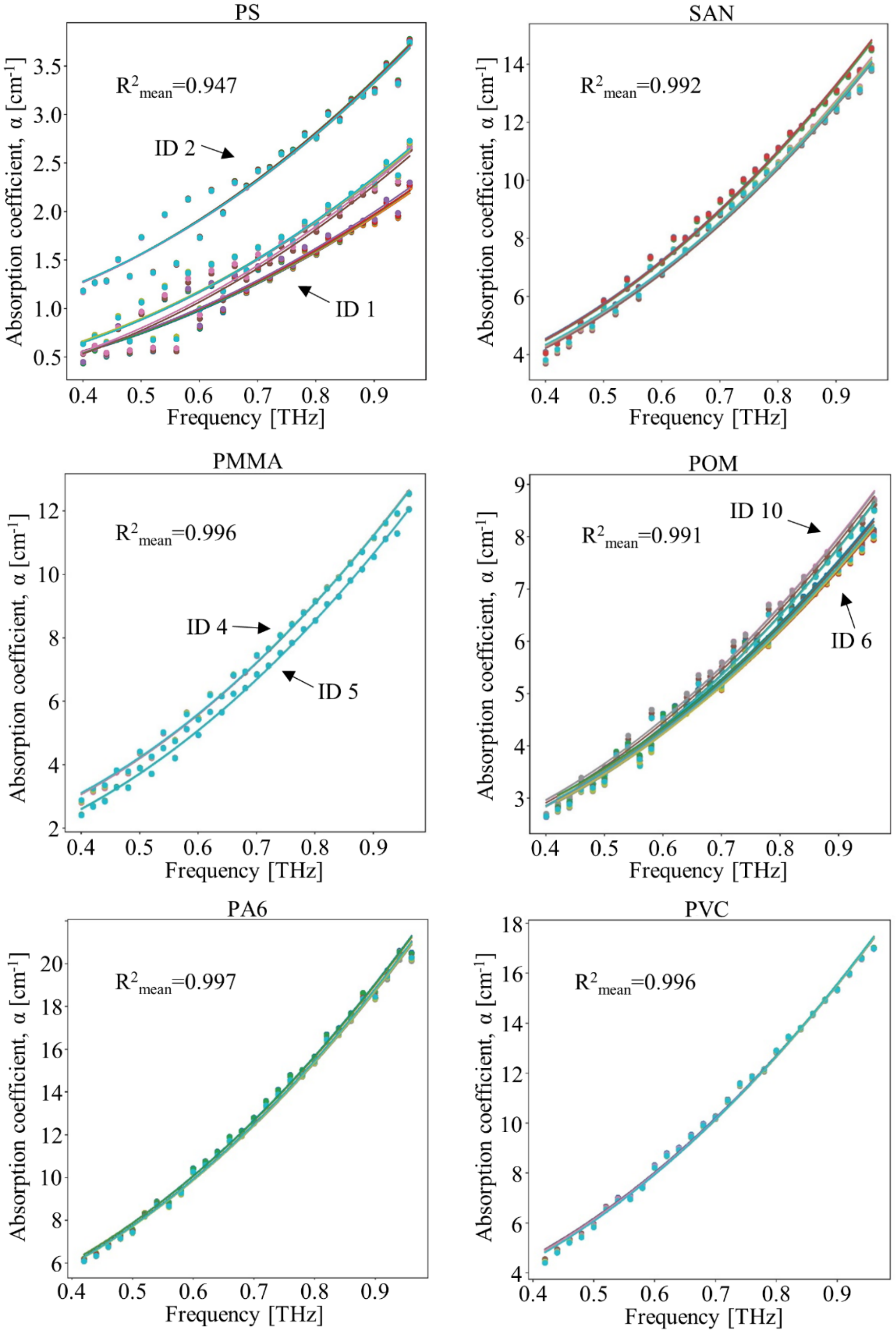

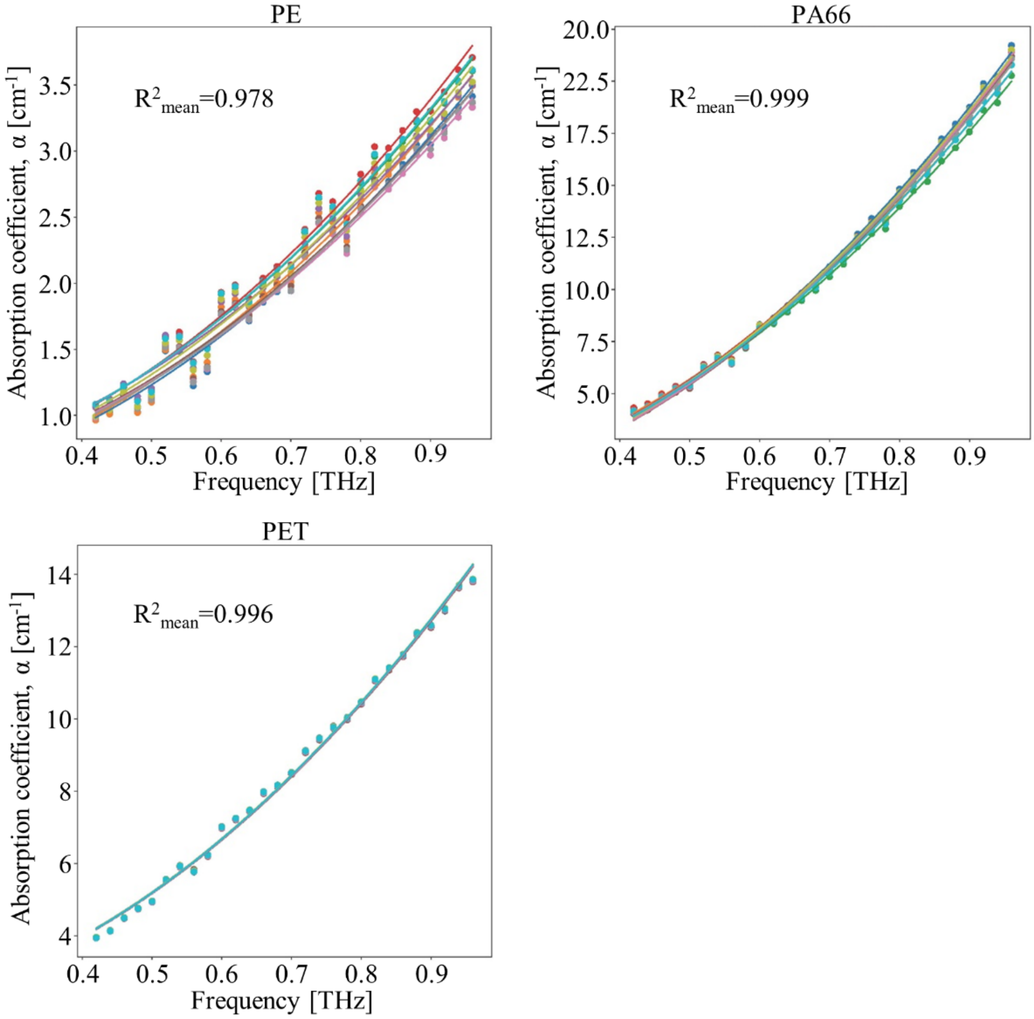

Table S2 shows the values of refractive index and fitting parameters for all samples in the range from 0.4 THz to 1.0 THz.

Table S2: Parameters extracted from all samples for the two-dimensional map shown in Fig. 5 in the frequency range from 0.4 THz to 1.0 THz: refractive index (n), absorption offset (α_0), absorption increase (β), and mean goodness-of-fit values (R^2).

ID	Abbr.	n	α_0 [cm^{-1}]	β	R^2
1	PS	1.5935 ± 0.0029	0.1990 ± 0.0468	2.399 ± 0.2473	0.9338
2	PS	1.6038 ± 0.0003	0.7678 ± 0.0063	3.175 ± 0.0259	0.9601
3	SAN	1.6378 ± 0.0026	13.107 ± 0.3028	2.276 ± 0.0778	0.9915
4	PMMA	1.5949 ± 0.0007	1.0794 ± 0.0231	12.51 ± 0.0351	0.9962
5	PMMA	1.5928 ± 0.0003	0.6103 ± 0.0062	12.38 ± 0.0067	0.9965
6	POM	1.6761 ± 0.0018	1.6596 ± 0.0410	7.626 ± 0.0563	0.9914
7	PA6	1.7395 ± 0.0007	19.775 ± 0.1186	0.825 ± 0.0443	0.9965
8	PVC	1.6037 ± 0.0005	16.815 ± 0.0970	1.920 ± 0.0656	0.9963
9	PE	1.5907 ± 0.0014	3.3950 ± 0.1348	0.449 ± 0.0356	0.9780
10	POM	1.6668 ± 0.0009	1.7479 ± 0.0474	7.036 ± 0.0571	0.9900
11	PA66	1.7998 ± 0.0031	22.711 ± 0.5983	-0.121 ± 0.1884	0.9985
12	PET	1.7284 ± 0.0005	13.481 ± 0.0274	1.821 ± 0.0108	0.9956

S4. Maximum absorption

The absorption for PE and PA66 together with the corresponding maximum obtainable absorption, α_{max}, for the dynamic range (DR) of our system is shown below. α_{max} is calculated as described by Jepsen and Fischer[40].

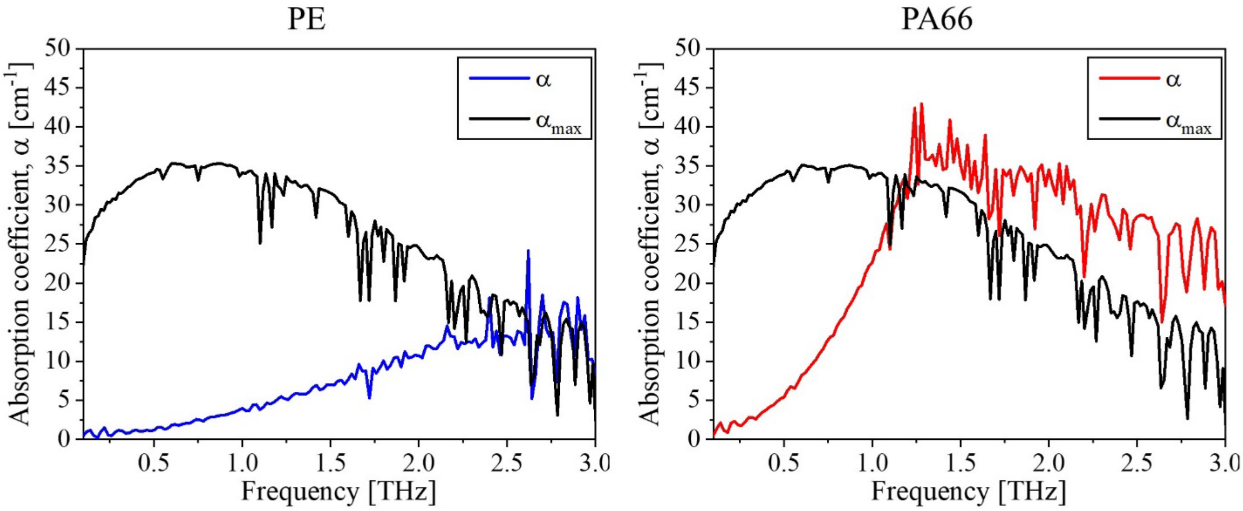

For the samples with the lowest absorption such as PE, the maximum frequency where the absorption can reliably be measured exceeds 2 THz under ambient conditions. For the most absorbent sample, PA66, the upper-frequency range of the measurable absorption is limited by the water absorption peak at 1.10 THz where α and α_{max} intersect.
